# Association of body mass index with incident tuberculosis in Korea

**DOI:** 10.1371/journal.pone.0195104

**Published:** 2018-04-18

**Authors:** Soo Jung Kim, Shinhee Ye, Eunhee Ha, Eun Mi Chun

**Affiliations:** 1 Division of Pulmonary and Critical Care Medicine, Department of Internal Medicine, College of Medicine, Ewha Womans University, Seoul, Republic of Korea; 2 Department of Occupational and Environmental Medicine, College of Medicine, Ewha Womans University, Seoul, Republic of Korea; University of British Columbia, CANADA

## Abstract

**Introduction:**

Overweight or obesity might be protective factors of tuberculosis (TB), but the evidence is inconclusive. The objective of study was to evaluate association between BMI and incident TB.

**Methods:**

The National Health Insurance database was used. Eligible participants were individuals aged 20–89 years without history of TB before 2007, and who underwent national health examinations between January 2002 and December 2006. The latest record of BMI was used as the exposure and categorized as follows: <18.5, 18.5–23, 23–25, 25–30, and ≥30 kg/m^2^. TB was defined as the first recorded diagnosis of TB, using ICD-10 between January 2007 and December 2013.

**Results:**

Among 301,081 individuals, 3,772 (1.26%) incident TB cases were detected. The incidence rate of the event was 19.65 per 10,000 person-years. After adjusting age, sex, household income, smoking status, alcohol use, and diabetes, incident TB was decreased as BMI was increased in an inverse dose-response relationship. However, when stratified by age and sex, BMI >30 kg/m^2^ did not show protective effect of TB in female under 50 years. Additionally, BMI >30 kg/m^2^ did not decrease incident TB in diabetics.

**Conclusion:**

Our study suggests that high BMI might be associated with decreased risk of TB. However, very high BMI did not reduce the risk of TB in young females or diabetics participants with in Korean population.

## Introduction

Low body mass index (BMI) is an important risk factor for the development of tuberculosis (TB) [1]. Additionally, there is growing evidence that high BMI is a protective factor of TB. Previous epidemiologic data reported that obesity was associated with reduced risk of active TB in an inverse dose-response relationship [2–5].

However, obesity has also been linked to diabetes mellitus (DM), which is an important risk factor for TB [6]. Furthermore, adipose tissue participates in inflammation and immunity, producing and releasing a variety of pro-inflammatory and anti-inflammatory factors that might influence susceptibility to infections [7]. In one study, BMI above 28 kg/m^2^ was independently associated with host susceptibility of TB in rural China [8]. Additionally, although an inverse logarithmic relationship between TB incidence and BMI was showed in another study, the relationship was uncertain at a BMI above 30 kg/m^2^ [3].

From the foregoing it is evident that the effect of overweight or obesity, especially a BMI exceeding 30 kg/m^2^, on the development of TB is inconclusive. Overweight and obesity might be a protective factor of TB, or a target for TB control. To provide clarity, we investigated the effect of BMI on TB in a Korean population, using a nationwide database from 2002 to 2013. We also performed a stratified analysis to explore whether any specific subgroups were more affected by BMI change.

## Methods

### Data source and study design

The present study was performed between January 1, 2002 and December 31, 2013, using data from the National Health Insurance (NHI) database, which includes a proportionate stratified random sample of individuals who visited hospitals under the NHI program that covers all legal residents of the Republic of Korea. The NHI database contains demographic information, including age, sex, household income, and district level address, and inpatient and outpatient medical care utilization information, including date of service, diagnosed disease based on Tenth Revision of the International Classification of Diseases (ICD-10), prescribed drugs, and medical or surgical procedures performed. The database also includes the health examination results of individuals who participated in free health examination services provided by the NHI. Individuals with lower income and disability have been reported to be less likely to participate in the free health examination compared with those with higher income and without disability. All data was completely anonymous before access.

Eligible participants were individuals who participated in national health examination services between January 1, 2002 and December 31, 2006, and who were aged 20–89 years of age in 2007. Individuals diagnosed as TB before January, 2007 and patients with human immunodeficiency virus (HIV) infection were excluded. Follow-up period was between January 1, 2007 and December 31, 2013.

This study approved by Institutional Review Board (IRB) of Ewha Womans University Medical Center (IRB number: 2016-08-038) and the NHI Service (research management number: NHIS-2016-2-202).

### Assessment of BMI

Of the health examinations data between January 1, 2002 and December 31, 2006, the latest record of BMI was used as the exposure of participants. Individuals were categorized into five BMI groups: <18.5, 18.5 to 22.9, 23.0 to 24.9, 25.0 to 29.9, and ≥30.0 kg/m^2^, as proposed by World Health Organization (WHO) for the Western-Pacific region [9] and by the Korean Society for the Study of Obesity [10, 11].

### TB outcome

Incident TB was defined as a first recorded diagnosis of TB based on ICD-10 code (A15.x-A19.x) during the follow-up period.

### Statistical analysis

To clarify the characteristics of study population, descriptive analyses were performed using the chi-square test for categorical variables and ANOVA test for continuous variables according to BMI level. The criterion for statistical significance was p < 0.05.

A multivariate Cox proportional hazards regression analysis was performed to evaluate the effects of BMI on the incidence of TB. Before the analysis, the assumption of proportionality was confirmed by Schoenfeld residuals. Multivariate Cox proportional hazards model was used adjusted by sex, household income (> and < 50^th^ percentile), smoking status (never smokers, former smokers, current smokers), alcohol use (never drinker, 2~3/month, 1~2/week, 3~4/week) and presence or absence of DM, which has been known as risk factors of TB [12–14]. Additional stratified analysis was performed to find whether specific subgroups were more affected by BMI change. All statistical analyses were performed using SAS version 9.3 software (SAS Institute Inc., Cary, NC).

## Results

### Study population

**Fig 1:**
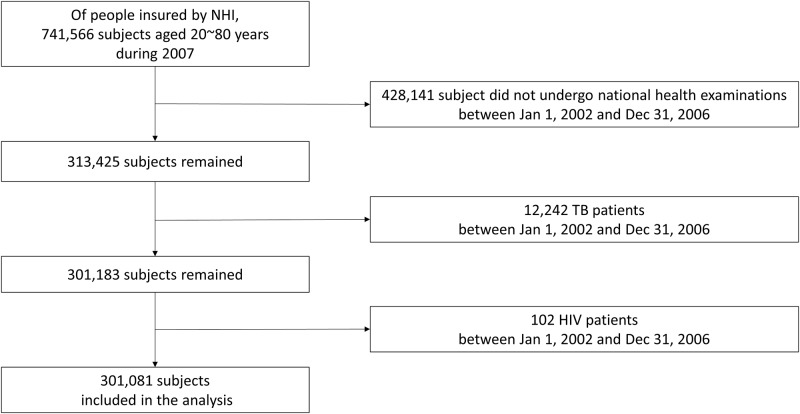
Study population. NHI indicates National Health Insurance; TB, tuberculosis; HIV, human immunodeficiency virus.

Between January 1, 2002 and December 31, 2006, 313,425 individuals underwent national health examinations (Fig 1). Among them, 12,242 subjects were excluded because they had a diagnosis of TB between January 1, 2002 and December 31, 2006. Additionally, 102 patients with HIV infection were excluded. Finally, 301,081 individuals aged 20–89 years in 2007 without TB before 2007 were enrolled. On the basis of BMI, 4.0%, 39.8%, 24.7%, 28.5%, and 3.0% of patients were in the BMI category of <18.5, 18.5–22.9, 23–24.9, 25–29.9, and ≥30 kg/m^2^, respectively. Baseline characteristics of cohort are presented in Table 1.

**Table 1:** Baseline characteristics of the cohort.

	BMI category (kg/m^2^)					
	<18.5 (N = 11,955)	18.5 to 22.9 (N = 119,813)	23.0 to 24.9 (N = 74,419)	25.0 to 29.9 (N = 85,806)	≥ 30.0 (N = 9,088)	*P-value*
Incidence of TB	257 (2.1)	1893 (1.6)	839 (1.1)	720 (0.8)	63 (0.7)	<0.001
Interval from BMI measurement to TB occurrence, years	4.1±2.4	4.2±2.3	4.3±2.3	4.3±2.3	4.2±2.2	0.8470
Age, years	41.7±17.1	46.3±14.5	49.9±13.2	50.6±13.0	48.7±14.0	<0.001
Sex						
Male	3754 (31.4)	55588 (46.4)	44171 (59.4)	53683 (62.6)	4946 (54.4)	<0.001
Female	8201(68.6)	64225 (53.6)	30248 (40.6)	32123 (37.4)	414 (45.6)	
Household income						
< 50^th^ percentile	5,172 (43.3)	44383 (37.0)	24510 (32.9)	27856 (32.5)	3,419 (37.6)	<0.001
≥ 50^th^ percentile	6,783 (56.7)	75430 (63.0)	49909 (67.1)	57950 (67.5)	5,669 (62.4)	
DM	146 (1.2)	2835 (2.4)	2636 (3.5)	3874 (4.5)	536 (5.9)	<0.001
Smoking status						
Never	8586 (77.8)	78747 (72.2)	45391 (68.2)	50644 (66.5)	5539 (67.3)	<0.001
Former	241 (2.2)	3866 (3.5)	3497 (5.3)	4322 (5.7)	357 (4.3)	
Current	2204 (20)	26501(24.3)	17627 (26.5)	21245 (27.9)	2330 (28.3)	
Alcohol use						
Never	6789 (58.5)	63254 (54.3)	37040 (51.3)	41942 (50.3)	4746 (53.7)	<0.001
2~3/month	2504 (21.6)	23412 (20.1)	13396 (18.5)	14664 (17.6)	1452 (16.4)	
1~2/week	1650 (14.2)	20158 (17.3)	14664 (20.3)	17657 (21.2)	1774 (20.1)	
3~4/week	375 (3.2)	6242 (5.4)	4972 (6.9)	6465 (7.8)	635 (7.2)	
Every day	282 (2.4)	3385 (2.9)	2202 (3.1)	2618 (3.1)	240 (2.7)	

Data are presented as number (%) or mean±SD

Abbreviations: BMI, body mass index; TB, tuberculosis; DM, diabetes mellitus.

### Effect of BMI on the risk of incident TB

Total person-years of follow-up was 1,919,106 person-year for the TB outcome. Overall mortality among different BMI groups was not different between high BMI groups (S1 Table). Among 301,081 individuals, 3,772 (1.26%) incident TB cases were detected. Types of TB are presented in S2 Table. The incidence rate of the event was 19.65 per 10,000 person-years. Development of TB was decreased as BMI was increased, being 2.1% for <18.5 kg/m2, 1.6% for 18.5–22.9 kg/m2, 1.1% for 23–24.9 kg/m2, 0.8% for 25–29.9 kg/m2, and 0.7% for ≥30 kg/m^2^ (*P* <0.001). There was no difference in the interval between BMI measurement and TB occurrence between BMI groups. Compared with those whose BMI was in the normal range (18.5–22.9kg/m^2^), a dose-dependent relationship between BMI and risk of TB was observed in the whole population after adjusting for age, sex, household income, smoking status, alcohol use, and diabetes, with the risk of TB was lower in overweight or obese individuals (Table 2). Risk of developing TB for participants with BMI ≥ 30kg/m^2^ was nearly 2.5-fold lower than those with normal BMI (adjusted hazard ratio [aHR], 0.40; 95% CI, 0.30–0.54).

**Table 2:** Effect of BMI on development of tuberculosis and subgroup analysis.

	BMI category (kg/m^2^)	Cases	Incidence rate (person-years)	Crude HR (95% CI)	*P-value*	aHR (95% CI)	*P-value*
All	<18.5	257	73645.7	1.43 (1.23–1.67)	<.0001	1.36 (1.16–1.61)	0.0002*
	18.5 to 22.9	1893	757521.9	1 (Reference)		1 (Reference)	
	23 to 24.9	839	477851.1	0.64 (0.59–0.71)	<.0001	0.63 (0.57–0.69)	<0.0001*
	25 to 29.9	720	551784.0	0.46 (0.41–0.51)	<.0001	0.45 (0.40–0.50)	<0.0001*
	≥ 30	63	58303.2	0.40 (0.30–0.53)	<.0001	0.40 (0.30–0.54)	<0.0001*
Age							
Age <50 years	<18.5	109	55001.6	1.23 (0.97–1.55)	0.0834	1.20 (0.93–1.54)	0.1541*
	18.5 to 22.9	781	480985.9	1 (Reference)		1 (Reference)	
	23 to 24.9	241	249777.3	0.58 (0.49–0.68)	<.0001	0.54 (0.45–0.65)	<0.0001*
	25 to 29.9	199	273823.1	0.43 (0.36–0.51)	<.0001	0.40 (0.33–0.49)	<0.0001*
	≥ 30	19	31991.8	0.39 (0.24–0.65)	0.0002	0.31 (0.17–0.54)	<0.0001*
Age ≥50 years	<18.5	148	18644.1	1.62 (1.33–1.98)	<.0001	1.46(1.18–1.82)	0.0007*
	18.5 to 22.9	1112	276535.9	1 (Reference)		1 (Reference)	
	23 to 24.9	598	228073.8	0.68 (0.61–0.76)	<.0001	0.69(0.61–0.78)	<0.0001*
	25 to 29.9	521	277961.0	0.47 (0.42–0.53)	<.0001	0.48(0.43–0.55)	<0.0001*
	≥ 30	44	26311.4	0.41 (0.29–0.58)	<.0001	0.48(0.34–0.68)	<0.0001*
Sex							
Male	<18.5	142	22699.7	1.76 (1.44–2.15)	<.0001	1.54(1.23–1.93)	0.0002^†^
	18.5 to 22.9	1072	352431.1	1 (Reference)		1 (Reference)	
	23 to 24.9	498	284072.4	0.58 (0.51–0.65)	<.0001	0.57 (0.50–0.65)	<0.0001^†^
	25 to 29.9	430	346054.6	0.43 (0.38–0.49)	<.0001	0.44 (0.39–0.51)	<0.0001^†^
	≥ 30	20	31821.1	0.28 (0.17–0.45)	<.0001	0.24 (0.14–0.42)	<0.0001^†^
Female	<18.5	115	50946.0	1.17 (0.92–1.47)	0.1961	1.15 (0.91–1.47)	0.2415^†^
	18.5 to 22.9	821	405090.8	1 (Reference)		1 (Reference)	
	23 to 24.9	341	193778.8	0.74 (0.64–0.86)	<.0001	0.74 (0.64–0.86)	<0.0001^†^
	25 to 29.9	290	205729.4	0.51 (0.43–0.60)	<.0001	0.48 (0.40–0.57)	<0.0001^†^
	≥ 30	43	26482.1	0.60 (0.42–0.85)	0.0047	0.61 (0.42–0.88)	0.0073^†^
DM							
Non-DM	<18.5	252	72838.6	1.43 (1.23–1.67)	<.0001	1.37(1.16–1.61)	0.0002^‡^
	18.5 to 22.9	1818	740707.2	1 (Reference)		1 (Reference)	
	23 to 24.9	779	461670.7	0.63 (0.57–0.69)	<.0001	0.62 (0.56–0.69)	<0.0001^‡^
	25 to 29.9	657	527883.4	0.45 (0.40–0.50)	<.0001	0.44 (0.39–0.49)	<0.0001^‡^
	≥ 30	53	54932.7	0.36 (0.26–0.49)	<.0001	0.35 (0.25–0.50)	<0.0001^‡^
With DM	<18.5	5	807.1	1.49 (0.54–4.12)	0.447	0.76 (0.18–3.12)	0.6978^‡^
	18.5 to 22.9	75	16814.7	1 (Reference)		1 (Reference)	
	23 to 24.9	60	16180.5	0.92 (0.62–1.35)	0.6605	0.82 (0.54–1.24)	0.3478^‡^
	25 to 29.9	63	23900.6	0.63 (0.43–0.93)	0.0199	0.64 (0.42–0.95)	0.0281^‡^
	≥ 30	10	3370.5	0.87 (0.43–1.76)	0.697	1.05 (0.51–2.15)	0.8901^‡^
Smoking status							
Never smoker	<18.5	147	53139.3	1.17 (0.95–1.44)	0.1317	1.19 (0.97–1.47)	0.0951^§^
	18.5 to 22.9	1165	496978.3	1 (Reference)		1 (Reference)	
	23 to 24.9	516	291286.7	0.65 (0.57–0.73)	<.0001	0.63 (0.56–0.71)	<0.0001^§^
	25 to 29.9	443	325135.6	0.45 (0.40–0.52)	<.0001	0.44 (0.39–0.50)	<0.0001^§^
	≥ 30	44	35411.1	0.42 (0.30–0.59)	<.0001	0.42 (0.30–0.60)	<0.0001^§^
Former smoker	<18.5	2	1474.3	0.57 (0.14–2.35)	0.434	0.59 (0.14–2.45)	0.4666^§^
	18.5 to 22.9	58	24723.3	1 (Reference)		1 (Reference)	
	23 to 24.9	36	22538.7	0.61 (0.38–0.98)	0.0426	0.61 (0.37–0.98)	0.0417^§^
	25 to 29.9	30	28120.0	0.46 (0.28–0.77)	0.0029	0.46 (0.28–0.76)	0.0027^§^
	≥ 30	2	2303.7	0.57 (0.14–2.34)	0.4309	0.57 (0.14–2.38)	0.4442^§^
Current smoker	<18.5	69	13595.0	1.86 (1.40–2.46)	<.0001	1.89 (1.43–2.50)	<0.0001^§^
	18.5 to 22.9	470	168653.7	1 (Reference)		1 (Reference)	
	23 to 24.9	192	113651.9	0.64 (0.53–0.77)	<.0001	0.63 (0.52–0.76)	<0.0001^§^
	25 to 29.9	158	137238.8	0.46 (0.38–0.57)	<.0001	0.46 (0.38–0.56)	<0.0001^§^
	≥ 30	11	15078.8	0.34 (0.18–0.66)	0.0015	0.34 (0.18–0.66)	0.0015^§^
Alcohol use							
Never	<18.5	152	41499.6	1.43 (1.17–1.75)	0.0005	1.37 (1.11–1.68)	0.0038^∥^
	18.5 to 22.9	1008	397568.0	1 (Reference)		1 (Reference)	
	23 to 24.9	473	236440.6	0.70 (0.62–0.80)	<.0001	0.69 (0.61–0.78)	<0.0001^∥^
	25 to 29.9	383	268232.4	0.47 (0.41–0.54)	<.0001	0.46 (0.40–0.53)	<0.0001^∥^
	≥ 30	38	30257.7	0.41 (0.28–0.60)	<.0001	0.42 (0.29–0.62)	<0.0001^∥^
2~3/month	<18.5	30	15840.3	1.04 (0.68–1.59)	0.8588	0.93 (0.59–1.47)	0.7671^∥^
	18.5 to 22.9	309	149967.2	1 (Reference)		1 (Reference)	
	23 to 24.9	108	87165.2	0.55 (0.42–0.7)	<.0001	0.51 (0.39–0.68)	<0.0001^∥^
	25 to 29.9	106	95196.5	0.47 (0.37–0.61)	<.0001	0.44 (0.33–0.58)	<0.0001^∥^
	≥ 30	7	9469.0	0.32 (0.13–0.77)	0.0107	0.27 (0.1–0.74)	0.0103^∥^
1~2/week	<18.5	30	10416.2	1.42 (0.92–2.17)	0.1102	1.20 (0.73–1.95)	0.4708^∥^
	18.5 to 22.9	284	129144.5	1 (Reference)		1 (Reference)	
	23 to 24.9	124	94726.4	0.54 (0.42–0.68)	<.0001	0.57 (0.44–0.73)	<0.0001^∥^
	25 to 29.9	120	114275.7	0.42 (0.33–0.54)	<.0001	0.45 (0.35–0.58)	<0.0001^∥^
	≥ 30	10	11423.7	0.42 (0.21–0.84)	0.0148	0.48 (0.23–0.97)	0.0397^∥^
3~4/week	<18.5	16	2156.8	2.57 (1.46–4.52)	0.0011	2.72 (1.51–4.92)	0.0009^∥^
	18.5 to 22.9	131	39530.1	1 (Reference)		1 (Reference)	
	23 to 24.9	63	31908.7	0.58 (0.41–0.83)	0.0023	0.61 (0.42–0.87)	0.0073^∥^
	25 to 29.9	42	41678.6	0.33 (0.22–0.48)	<.0001	0.31 (0.21–0.48)	<0.0001^∥^
	≥ 30	4	4076.3	0.47 (0.17–1.27)	0.1336	0.38 (0.12–1.20)	0.1000^∥^
Every day	<18.5	21	1572.7	2.01 (1.13–3.59)	0.0175	1.73 (0.91–3.29)	0.0975^∥^
	18.5 to 22.9	98	20463.9	1 (Reference)		1 (Reference)	
	23 to 24.9	39	13908.9	0.72 (0.47–1.08)	0.1135	0.67 (0.42–1.05)	0.0785^∥^
	25 to 29.9	43	16694.7	0.67 (0.44–1.01)	0.0571	0.62 (0.39–0.97)	0.0367^∥^
	≥ 30	2	1502.4	0.50 (0.12–2.03)	0.3283	0.54 (0.13–2.22)	0.3915^∥^

Abbreviations: BMI, body mass index; DM, diabetes mellitus.

*Adjusted by sex, household income, smoking status, alcohol use, and diabetes

^†^Adjusted by age, household income, smoking status, alcohol use, and diabetes

^‡^Adjusted by age, sex, household income, smoking status, alcohol use

^§^Adjusted by age, sex, household income, alcohol use, and diabetes

^∥^Adjusted by age, sex, household income, smoking status, and diabetes

### Subgroup analysis according to age and sex

When stratified according to age < or ≥50 years of age, overweight and obesity was associated with reduced a risk of incident TB. For subjects <50 years of age, the HR decreased as BMI increased. However, in individuals ≥50 of age and older, the HR was not different between participants with BMI 25 to 29.9 kg/m^2^ (aHR, 0.48; 95% CI, 0.43–0.55) and ≥30kg/m^2^ (aHR, 0.48, 95% CI, 0.34–0.68). Additionally, among same BMI categories, the risk of development of TB in individuals <50 years of age was lower compared those 50 years of age and older.

When stratified by sex, the risk of TB decreased as BMI increased, and those with BMI >30kg/m^2^ had the lowest risk of TB in the male cohort. Compared to males with normal BMI, there was 76% risk reduction effect of incident TB in males with BMI >30 kg/m^2^ (aHR, 0.24; 95% CI, 0.14–0.42). In contrast, in the female cohort, those with BMI 25–29.9kg/m^2^ had the lowest risk of TB (aHR, 0.48; 95% CI, 0.40–0.57).

When stratified by both age and sex, inverse dose–response relationship was evident between BMI and risk of TB both in males and females above 50 years of age (Table 3). However, in female <50 years of age, those with BMI >30 kg/m^2^ did not show a protective effect of TB (aHR, 0.77; 95% CI, 0.36–1.63).

**Table 3:** Effect of BMI: Subgroup analysis by sex and age.

	BMI category (kg/m^2^)	Cases	Incidence rate (person-years)	Crude HR (95% CI)	*P-value*	aHR (95% CI)*	*P-value*
Male							
Age <50 years	<18.5	50	13473.6	2.07 (1.48–2.9)	<.0001	1.77 (1.22–2.56)	0.0028
	18.5 to 22.9	400	222239.6	1 (Reference)		1 (Reference)	
	23 to 24.9	172	171368.7	0.53 (0.43–0.65)	<.0001	0.53 (0.42–0.65)	<0.0001
	25 to 29.9	150	212613.6	0.37 (0.30–0.46)	<.0001	0.37 (0.29–0.47)	<0.0001
	≥ 30	9	23528.0	0.24 (0.12–0.49)	<.0001	0.17 (0.07–0.41)	<0.0001
Age ≥50 years	<18.5	92	9226.1	1.64 (1.27–2.10)	0.0001	1.44 (1.08–1.91)	0.0122
	18.5 to 22.9	672	130191.5	1 (Reference)		1 (Reference)	
	23 to 24.9	326	112703.6	0.60 (0.52–0.70)	<.0001	0.60 (0.51–0.71)	<0.0001
	25 to 29.9	280	133441.0	0.46 (0.39–0.54)	<.0001	0.49 (0.42–0.58)	<0.0001
	≥ 30	11	8293.1	0.31 (0.16–0.60)	0.0005	0.33 (0.16–0.66)	0.0018
Female							
Age <50 years	<18.5	59	41528.0	0.93 (0.68–1.29)	0.6774	0.93 (0.67–1.30)	0.6718
	18.5 to 22.9	381	258746.3	1 (Reference)		1 (Reference)	
	23 to 24.9	69	78408.6	0.61 (0.46–0.82)	0.0009	0.59 (0.44–0.80)	0.0007
	25 to 29.9	49	61209.5	0.53 (0.38–0.75)	0.0003	0.52 (0.37–0.75)	0.0004
	≥ 30	10	8463.8	0.81 (0.40–1.63)	0.5494	0.77 (0.36–1.63)	0.4957
Age ≥50 years	<18.5	56	9418.0	1.53 (1.10–2.13)	0.0114	1.51 (1.07–2.13)	0.0185
	18.5 to 22.9	440	146344.5	1 (Reference)		1 (Reference)	
	23 to 24.9	272	115370.2	0.80 (0.68–0.96)	0.0131	0.81 (0.68–0.97)	0.0203
	25 to 29.9	241	144520.0	0.52 (0.43–0.62)	<.0001	0.48 (0.40–0.58)	<0.0001
	≥ 30	33	18018.4	0.56 (0.37–0.85)	0.0066	0.59 (0.39–0.89)	0.0126

Abbreviations: BMI, body mass index.

*Adjusted by household income, smoking status, alcohol use, and diabetes

### Subgroup analysis according to diabetes

Subgroup analysis was performed according to presence or absence of DM. In participants without diabetes, risk of TB was decreased as BMI increased. However, in participants with DM, the risk of tuberculosis was significantly lower in those with BMI 25–29.9kg/m^2^, compared with those with normal BMI. A BMI >30 kg/m^2^ was not a protective factor of incident TB in participants with DM (aHR, 1.05; 95% CI, 0.51–2.15).

### Subgroup analysis according to smoking status and alcohol use

Stratification based on smoking status showed that risk of TB was decreased as BMI increased in all subgroups. When stratified by both sex and smoking status, risk of developing TB for male never-smoker with BMI ≥30 kg/m^2^ was nearly 5-fold lower than those with normal BMI (aHR, 0.19; 95% CI, 0.08–0.41) (Table 4, S3 Table). In ever-smoker males, risk of developing TB for those with BMI ≥30 kg/m^2^ was nearly 3-fold lower than those with normal BMI (aHR, 0.32; 95% CI, 0.17–0.62). Ever-smoker females with low BMI <18.5 kg/m^2^ had the highest risk for developing TB (aHR, 4.16; 95% CI, 1.96–8.85).

**Table 4:** Effect of BMI: Subgroup analysis by sex and smoking status.

	BMI category (kg/m^2^)	Cases	Incidence rate (person-years)	Crude HR (95% CI)	*P-value*	aHR (95% CI)*	*P-value*
Male							
Never-smokers	<18.5	83	10029.8	1.95 (1.49–2.56)	<.0001	1.95(1.48–2.58)	<0.0001
	18.5 to 22.9	576	173654.0	1 (Reference)		1 (Reference)	
	23 to 24.9	281	152939.2	0.56 (0.47–0.65)	<.0001	0.54 (0.46–0.64)	<0.0001
	25 to 29.9	246	186246.4	0.41 (0.34–0.49)	<.0001	0.41 (0.35–0.49)	<0.0001
	≥ 30	10	15594.0	0.24 (0.12–0.48)	<.0001	0.19 (0.08–0.41)	<0.0001
Ever-smokers	<18.5	59	12669.9	1.57 (1.16–2.12)	0.0036	1.57 (1.16–2.12)	0.0036
	18.5 to 22.9	496	178777.1	1 (Reference)		1 (Reference)	
	23 to 24.9	217	131133.1	0.61 (0.51–0.73)	<.0001	0.61 (0.51–0.73)	<0.0001
	25 to 29.9	184	159808.2	0.46 (0.38–0.55)	<.0001	0.46 (0.38–0.55)	<0.0001
	≥ 30	10	16227.1	0.32 (0.17–0.63)	0.0008	0.32 (0.17–0.62)	0.0007
Female							
Never-smokers	<18.5	103	48546.6	1.04 (0.81–1.34)	0.7357	1.06 (0.82–1.36)	0.6594
	18.5 to 22.9	789	390490.9	1 (Reference)		1 (Reference)	
	23 to 24.9	330	188721.3	0.74 (0.64–0.86)	<.0001	0.74 (0.64–0.86)	<0.0001
	25 to 29.9	286	200178.9	0.51 (0.44–0.60)	<.0001	0.49 (0.42–0.58)	<0.0001
	≥ 30	40	25326.7	0.58 (0.40–0.83)	0.0034	0.60 (0.42–0.87)	0.0068
Ever-smokers	<18.5	12	2399.4	4.14 (1.95–8.76)	0.0002	4.16 (1.96–8.85)	0.0002
	18.5 to 22.9	32	14599.9	1 (Reference)		1 (Reference)	
	23 to 24.9	11	5057.4	0.81 (0.32–2.04)	0.6498	0.80 (0.31–2.02)	0.6291
	25 to 29.9	4	5550.6	0.34 (0.10–1.18)	0.0895	0.34 (0.10–1.17)	0.0864
	≥ 30	3	1155.4	1.24 (0.29–5.41)	0.7731	1.23 (0.28–5.36)	0.7878

Abbreviations: BMI, body mass index.

*Adjusted by age, household income, alcohol use, and diabetes

Stratification based on alcohol use was performed. There were trends to decreased risk of TB with increased BMI in all subgroups.

## Discussion

Our results suggest that higher BMI might be a protective factor of the development of TB, with inverse dose-response relationship between BMI and incident TB. However, very high BMI (≥30 kg/m^2^) did not reduce the risk of incident TB in young females and participants with DM in this Korean population.

Previous epidemiologic data support the view that obesity might be a protective factor of incident TB [2–5]. An association between BMI and incident TB has been reported in diverse populations. Leung et al. [2] reported that obesity is associated with a lower risk of active TB in elderly people >65 years of age in Hong Kong. Cegielski et al. [4] reported that incidence rate and risk of incident TB were decreased as BMI increased in a United States cohort. Additionally, they reported that persons who were overweight, had thick fat, or had large muscles had only one-third to one-fifth the risk of TB as people with normal values for these measures. A systematic review reported a log-linear inverse relationship between TB incidence and BMI, within the BMI range of 18.5–30 kg/m^2^ [3].

The protective effect of high BMI on TB might be due to altered immunity. Adipocytes release adipocytokines, which cause the interaction between adipose tissue, inflammation, and immunity [15]. Roth et al. [16] reported that individuals with higher visceral fat release higher amount of tumor necrosis factor (TNF) and other pro-inflammatory markers, which provide an advantage to protect TB. TNF-α is a critical immune mediator in protection against and pathology of TB [17], which has been illustrated by patients who receive TNF antagonist [18]. Furthermore, the adipocyte-macrophage groupings behave like an endocrine gland, releasing TNF, leptin, and other messenger molecules into the circulation, which is capable of influencing host cells and microbes throughout the body, and perhaps providing a protective effect of TB [19]. Anuradha et al. [20] also suggested that the modulation of protective and regulatory cytokines might underlie the protective effect of higher BMI against the development of active TB. Additionally, human visceral adipose tissue has a larger quantity of T lymphocytes per gram in obese subjects [21]. Because the adaptive immune response mediated by T cells is critical for control of TB infection in humans [22], T lymphocytes in adipose tissue might affect development of TB.

However, it is uncertain whether very high BMI, especially BMI ≥ 30 kg/m^2^, is protective factors of incident TB. A log-linear inverse relationship between TB incidence and BMI was reportedly uncertain for BMI ≥30 kg/m^2^ [3]. Additionally, Zhang et al. [8] reported that BMI exceeding 28 kg/m^2^ was independently associated with host susceptibility of TB in rural China.

Our study suggests that high BMI is associated with a low risk of incident TB. However, not all subgroups showed this inverse dose-response relationship between BMI and risk of incident TB. Interestingly, there was a sex-specific effect of BMI on incident TB. In males, incident TB was more sensitive to BMI, compared with females. The risk of incident TB decreased as BMI increased, and the lowest risk group was males with BMI >30 kg/m^2^. In contrast, although females with high BMI displayed a reduced risk of incident TB, the lowest risk group was those with BMI of 25–29.9 kg/m^2^. Additionally, when we stratified for sex and age, BMI >30 kg/m^2^ did not reduce incident TB in females under 50 years of age; some may be premenopausal women. The risk of incident TB in females ≥50 years of age, who are expected to be postmenopausal, was similar to males concerting sensitivity to BMI.

The results indicate that the effect of BMI on risk reduction of incident TB might be sensitive in males and postmenopausal females. This age- and sex-specific difference of BMI effect might be caused by hormonal differences. It has been reported that testosterone, the primary male sex hormone, impairs macrophage activation and could play a detrimental role in TB [23]. Bini et al. also demonstrated that male mice are more susceptible to TB than female mice, and that this finding was prevented by castration, suggesting that testosterone could be a TB susceptibility factor. Since increased fat mass has been associated with low testosterone [24, 25], high BMI could decrease the risk of incident TB in males. Additionally, in postmenopausal women, as ovarian production of sex hormones declines, the main source of these hormones is from the adrenal glands, with peripheral conversion into their active form in adipose tissue [26]. This might cause different effect of BMI on incident TB between females under and over 50 years of age. Furthermore, Ilavska et al. showed that a sex-dependent association between BMI and white blood cell subpopulation counts in peripheral blood, which reflecting strong association between BMI and human immune response [27]. This study can provide one of explanation of sex-specific BMI effect on incident TB in our study.

Our study also showed that the effect of BMI on reduced risk of incident TB was different between individual with and without DM. In diabetics, high BMI was not a protective factor of incident TB. DM itself was a risk factor of TB, and increased as BMI increased. Glycemic control level of each individual also might affect the results. Leung et al. reported that diabetic subjects with hemoglobin A1c <7% at enrollment were not at increased risk [28].

The strength of our study is that it was nationwide population-based study including the largest number of study population compared with previous studies. Furthermore, we performed stratification analyses based on various subgroups. Additionally, there was time lag between record of latest BMI and diagnosis of TB based on ICD-10 code. Moreover, overall mortality between high BMI groups was evaluated in our study. It could be argued that difference of overall mortality among different BMI groups would be a confounding factor as high BMI is associated with increased mortality [11]. However, we demonstrated that overall mortality between high BMI groups was not different (S1 Table).

However, there were some limitations. First of all, this study was retrospective study. In addition, BMI does not distinguish between adipose fat, muscle, bone, and water. Further study using methods that could distinguish fat would be instructive. Third, we could not evaluate hormonal levels or HbA1c because we used insurance claim data. Finally, we could not clarify exact underlying pathophysiology of the effect of BMI on reduced risk of incident TB. More research is needed to elucidate the pathophysiology, focusing on TB and alteration of immune response according to BMI.

## Conclusion

In conclusion, overweight and obesity might be protective factors of the development of TB, with an inverse dose-response relationship between BMI and incident TB. However, very high BMI of ≥30kg/m^2^ did not reduced risk of TB in some subgroup including young female and participants with DM in this Korean population.

## Supporting information

S1 Table Representation for comparison of proportion of occurrence of death by BMI. (DOCX)

S2 Table Proportion of tuberculosis subtype. (DOCX)

S3 Table Subgroup analysis by sex and smoking status. (DOCX)
