# Correction: Association of body mass index with incident tuberculosis in Korea

**DOI:** 10.1371/journal.pone.0199065

**Published:** 2018-06-08

**Authors:** The PLOS ONE Staff

The first two authors, Soo Jung Kim and Shinhee Ye, should be noted as contributing equally to this work. The publisher apologizes for the error.
