# Supplementary material for: Association of body mass index with incident tuberculosis in Korea
Source: PLoS One. 2018 Apr 18;13(4):e0195104. doi: 10.1371/journal.pone.0195104 (PMC5906015; doi:10.1371/journal.pone.0195104)
Supplement: S1 Table — (DOCX) [file pone.0195104.s001.docx]

**S1 Table. Representation for comparison of proportion of occurrence of death by BMI**

| **BMI category (kg/m^2^)** | **Total**  **(n=301,081)** | **Censoring**  **(n=287,610)** | **Tuberculosis**  **(n=3,772)** | **Death**  **(n=9,699)** |
| --- | --- | --- | --- | --- |
| <18.5 | 11,955 (100) | 11059 (92.5) | 257 (2.2) | 639 (5.4) |
| 18.5 to 22.9 | 119,813 (100) | 113861 (95.0) | 1893 (1.6) | 4059 (3.4) |
| 23 to 24.9 | 74,419 (100) | 71388 (95.9) | 839 (1.1) | 2192 (3.0) |
| 25 to 29.9 | 85,806 (100) | 82558 (96.2) | 720 (0.8) | 2528 (3.0) |
| ≥ 30 | 9,088 (100) | 8744 (96.2) | 63 (0.7) | 281 (3.1) |

Data are presented as n (%)
