# Supplementary material for: Association of body mass index with incident tuberculosis in Korea
Source: PLoS One. 2018 Apr 18;13(4):e0195104. doi: 10.1371/journal.pone.0195104 (PMC5906015; doi:10.1371/journal.pone.0195104)
Supplement: S2 Table — (DOCX) [file pone.0195104.s002.docx]

**S2 Table. Proportion of tuberculosis subtype**

| **ICD-10 code** | **Disease name** | **N (%)** |
| --- | --- | --- |
| A15 | Respiratory tuberculosis, bacteriologically and histologically confirmed | 1285 (34.1) |
| A16 | Respiratory tuberculosis, not confirmed bacteriologically or histologically | 2051 (54.4) |
| A17 | Tuberculosis of nervous system | 17 (0.5) |
| A18 | Tuberculosis of other organs | 401 (10.6) |
| A19 | Miliary tuberculosis | 18 (0.5) |
| Total |  | 3772 (100) |
