# Supplementary material for: Association of body mass index with incident tuberculosis in Korea
Source: PLoS One. 2018 Apr 18;13(4):e0195104. doi: 10.1371/journal.pone.0195104 (PMC5906015; doi:10.1371/journal.pone.0195104)
Supplement: S3 Table — (DOCX) [file pone.0195104.s003.docx]

**S3 Table. Subgroup analysis by sex and smoking status**

|  | **BMI category (kg/m^2^)** | **Cases** | **Incidence rate (person-years)** | **Unadjusted HR (95% CI)** | ***p* Value** | **aHR (95% CI) ^*^** | ***p* Value** |
| --- | --- | --- | --- | --- | --- | --- | --- |
| Male |  |  |  |  |  |  |  |
| Never | <18.5 | 54 | 7724.7 | 1.57(1.12-2.21) | 0.0085 | 1.55(1.10-2.19) | 0.0119 |
|  | 18.5 to 22.9 | 426 | 129557.3 | 1 (Reference) |  | 1 (Reference) |  |
|  | 23 to 24.9 | 206 | 113764.8 | 0.52(0.43-0.63) | <.0001 | 0.52(0.43-0.63) | <.0001 |
|  | 25 to 29.9 | 186 | 136777.6 | 0.42(0.34-0.51) | <.0001 | 0.42(0.34-0.51) | <.0001 |
|  | ≥ 30 | 5 | 11598.1 | 0.16(0.06-0.43) | 0.0003 | 0.16(0.06-0.42) | 0.0002 |
| Former | <18.5 | 2 | 1048.0 | 0.66(0.16-2.74) | 0.5681 | 0.65(0.16-2.72) | 0.5599 |
|  | 18.5 to 22.9 | 54 | 22316.0 | 1 (Reference) |  | 1 (Reference) |  |
|  | 23 to 24.9 | 35 | 21759.7 | 0.60(0.37-0.98) | 0.0431 | 0.60(0.37-0.98) | 0.0428 |
|  | 25 to 29.9 | 30 | 27398.6 | 0.47(0.28-0.79) | 0.0041 | 0.47(0.28-0.78) | 0.0038 |
|  | ≥ 30 | 1 | 2159.2 | 0.30(0.04-2.14) | 0.2274 | 0.30(0.04-2.19) | 0.2349 |
| Current | <18.5 | 57 | 11621.9 | 1.66(1.22-2.26) | 0.0014 | 1.66(1.22-2.27) | 0.0013 |
|  | 18.5 to 22.9 | 442 | 156461.1 | 1 (Reference) |  | 1 (Reference) |  |
|  | 23 to 24.9 | 182 | 109373.5 | 0.63(0.52-0.76) | <.0001 | 0.62(0.51-0.76) | <.0001 |
|  | 25 to 29.9 | 154 | 132409.6 | 0.46(0.38-0.57) | <.0001 | 0.46(0.38-0.57) | <.0001 |
|  | ≥ 30 | 9 | 14067.8 | 0.33(0.16-0.67) | 0.0021 | 0.33(0.16-0.66) | 0.0018 |
| Female |  |  |  |  |  |  |  |
| Never | <18.5 | 93 | 45414.6 | 1.02(0.79-1.32) | 0.8698 | 1.03(0.79-1.33) | 0.8437 |
|  | 18.5 to 22.9 | 739 | 367421.0 | 1 (Reference) |  | 1 (Reference) |  |
|  | 23 to 24.9 | 310 | 177521.9 | 0.74(0.63-0.86) | <.0001 | 0.74(0.64-0.86) | 0.0001 |
|  | 25 to 29.9 | 257 | 188358.0 | 0.48(0.41-0.57) | <.0001 | 0.48(0.41-0.57) | <.0001 |
|  | ≥ 30 | 39 | 23813.0 | 0.59(0.40-0.85) | 0.0052 | 0.59(0.40-0.85) | 0.0053 |
| Former | <18.5 | 0 | 426.4 | - | 0.9982 |  | 0.9990 |
|  | 18.5 to 22.9 | 4 | 2407.2 | 1 (Reference) |  | 1 (Reference) |  |
|  | 23 to 24.9 | 1 | 779.0 | 0.53(0.05-5.35) | 0.5939 | 0.65(0.06-7.32) | 0.7234 |
|  | 25 to 29.9 | 0 | 721.3 | - | 0.9962 |  | 0.9975 |
|  | ≥ 30 | 1 | 144.5 | 17.09 (1.46-200.6) | 0.0239 | 10.57(0.32-349.93) | 0.1866 |
| Current | <18.5 | 12 | 1973.0 | 4.80(2.20-10.48) | <.0001 | 4.72(2.16-10.32) | 0.0001 |
|  | 18.5 to 22.9 | 28 | 12192.6 | 1 (Reference) |  | 1 (Reference) |  |
|  | 23 to 24.9 | 10 | 4278.4 | 0.83(0.30-2.30) | 0.7244 | 0.83(0.30-2.29) | 0.7213 |
|  | 25 to 29.9 | 4 | 4829.2 | 0.42(0.12-1.46) | 0.1701 | 0.40(0.12-1.41) | 0.1552 |
|  | ≥ 30 | 2 | 1010.9 | 0.73(0.10-5.60) | 0.765 | 0.75(0.1-5.74) | 0.7822 |

Abbreviations: BMI, body mass index.

**^*^**Adjusted by age, household income, alcohol use, and diabetes
